# The Non-coding Mammary Carcinoma Susceptibility Locus, *Mcs5c*, Regulates *Pappa* Expression via Age-Specific Chromatin Folding and Allele-Dependent DNA Methylation

**DOI:** 10.1371/journal.pgen.1006261

**Published:** 2016-08-18

**Authors:** Amanda N. Henning, Jill D. Haag, Bart M. G. Smits, Michael N. Gould

**Affiliations:** 1 Department of Oncology, McArdle Laboratory for Cancer Research, University of Wisconsin–Madison School of Medicine and Public Health, Madison, Wisconsin, United States of America; 2 Department of Pathology and Laboratory Medicine, Hollings Cancer Center, Medical University of South Carolina, Charleston, South Carolina, United States of America; National Cancer Institute, UNITED STATES

## Abstract

In understanding the etiology of breast cancer, the contributions of both genetic and environmental risk factors are further complicated by the impact of breast developmental stage. Specifically, the time period ranging from childhood to young adulthood represents a critical developmental window in a woman’s life when she is more susceptible to environmental hazards that may affect future breast cancer risk. Although the effects of environmental exposures during particular developmental Windows of Susceptibility (WOS) are well documented, the genetic mechanisms governing these interactions are largely unknown. Functional characterization of the Mammary Carcinoma Susceptibility 5c, *Mcs5c*, congenic rat model of breast cancer at various stages of mammary gland development was conducted to gain insight into the interplay between genetic risk factors and WOS. Using quantitative real-time PCR, chromosome conformation capture, and bisulfite pyrosequencing we have found that *Mcs5c* acts within the mammary gland to regulate expression of the neighboring gene *Pappa* during a critical mammary developmental time period in the rat, corresponding to the human young adult WOS. Pappa has been shown to positively regulate the IGF signaling pathway, which is required for proper mammary gland/breast development and is of increasing interest in breast cancer pathogenesis. *Mcs5c*-mediated regulation of *Pappa* appears to occur through age-dependent and mammary gland-specific chromatin looping, as well as genotype-dependent CpG island shore methylation. This represents, to our knowledge, the first insight into cellular mechanisms underlying the WOS phenomenon and demonstrates the influence developmental stage can have on risk locus functionality. Additionally, this work represents a novel model for further investigation into how environmental factors, together with genetic factors, modulate breast cancer risk in the context of breast developmental stage.

## Author Summary

A woman’s lifetime risk of developing breast cancer is affected by both genetic and environmental risk factors that can be further exacerbated by breast developmental stage. Time periods conferring increased risk are referred to as Windows of Susceptibility (WOS) and, generally speaking, the molecular mechanisms responsible for their effect on breast cancer risk are unknown. Our work presented here on the characterization of the rat Mammary Carcinoma Susceptibility 5c, *Mcs5c*, locus has identified a region within *Mcs5c* that interacts with the neighboring gene, *Pappa*, in an age-dependent manner to influence gene expression via genotype-dependent DNA methylation. Importantly, *Mcs5c*-mediated gene regulation occurs specifically within a WOS, and these finding represent the first identified molecular mechanisms by which a WOS influences the ability of a locus to affect mammary/breast cancer risk. This work highlights the importance developmental stage can have on genetic risk factor function, and we anticipate that the *Mcs5c* locus will serve as a model for future studies on WOS in combination with genetic and environmental risk factors.

## Introduction

In the United States, breast cancer is the most frequently diagnosed cancer and second leading cause of cancer death among women [1]. Its etiology is complex, consisting of the interaction of both genetic and environmental risk factors whose contribution to overall risk can vary depending on the developmental context of the individual. In general, time periods in which women are more susceptible to initiating events affecting their long term breast cancer risk are broadly referred to as Windows of Susceptibility (WOS) [2]. In humans, the best documentation of a WOS can be found in studies of radiation exposure in women. Women exposed to radiation between 0 and 30 years of age during either the atomic bombings of Japan or for the treatment of Hodgkin’s lymphoma had an increased risk of developing breast cancer later in life compared to women >30 years of age at time of exposure [3,4]. This time period, therefore, represents one of the WOS, and encompasses ages spanning childhood, adolescence, and young adulthood in women. Animals studies performed in rats to model the human WOS phenomenon [5] further suggest the existence of at least two mechanistically distinct susceptibility windows within the larger human WOS, namely, the sexually immature WOS (iWOS) and the adolescent WOS (aWOS). This division of the WOS is most evident in work by Ariazi *et al*. [6] on a carcinogen-inducible model of breast cancer, where administration to developmentally immature (3 week) and adolescent-aged (7 week) rats resulted in differential carcinoma development depending on age of administration and the carcinogen used. Additionally, although over 80 genetic loci affecting breast cancer susceptibility have been identified in human genome-wide association studies (GWAS) [summarized in 7], their function in relation to developmental stages has not been characterized. In general, while the effects of window specific exposures are well documented, the cellular mechanisms responsible for their function and governing their interactions with environmental and genetic risk factors are poorly understood.

**Fig 1:**
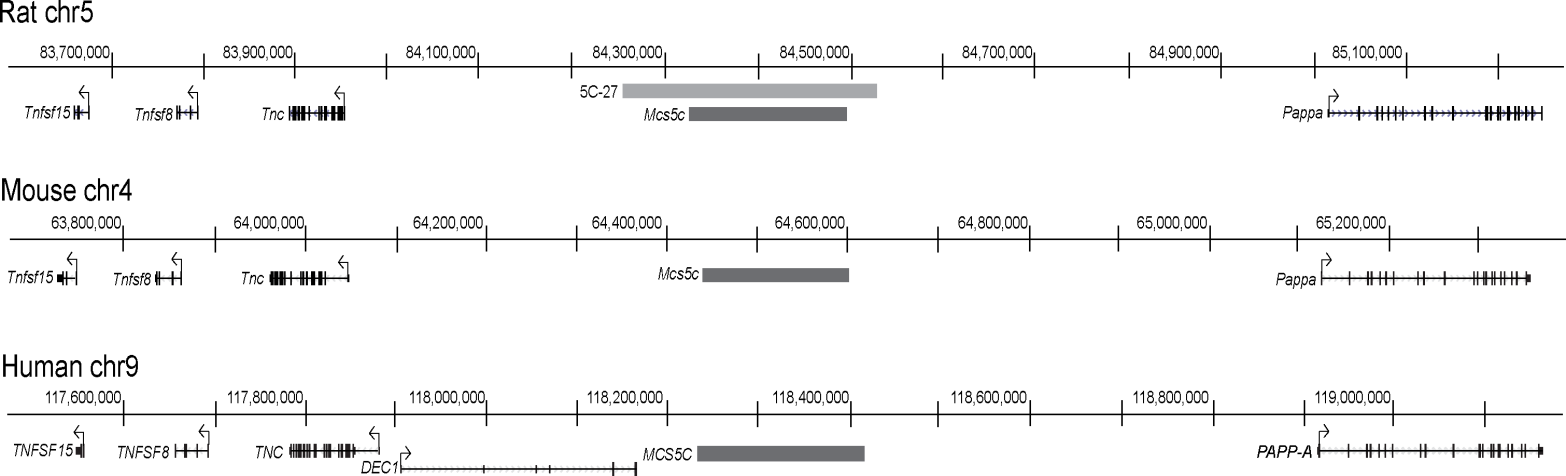
Comparative genomic map of the *Mcs5c* locus. Map coordinates for the *Mcs5c* locus (dark gray bar) and neighboring genes are depicted for the rat (UCSC Genome Browser, Mar. 2012, rn5), mouse (UCSC, Dec. 2011, mm10) and human (UCSC, Feb. 2009, hg19) genomes, and were determined based on sequence homology. The WKy-homozygous genomic region of congenic line 5C-27 is shown in light gray relative to the rat *Mcs5c* locus. This congenic line carries the smallest defined region of the locus (275kb) and was the resistant line used for all *Mcs5c* experiments. The remainder of the 5C-27 line is WF-homozygous (chr–chromosome; *Tnfsf15/TNFSF15* –Tumor Necrosis Factor (Ligand) Superfamily, Member 15; *Tnfsf8/TNFSF8* –Tumor Necrosis Factor (Ligand) Superfamily, Member 8; *Tnc/TNC*–Tenascin C; *DEC1* –Deleted in Esophageal Cancer 1; *Pappa/PAPP-A*–Pregnancy-Associated Plasma Protein A).

To begin to understand the complex interactions between WOS, genetics, and the environment, we turned to a comparative genomics approach, utilizing a rat model of breast cancer. The rat is an excellent model for this type of study, as not only does its mammary gland and mammary tumor development mimic that of the human condition [8], but, as previously mentioned, it too displays the WOS phenomenon [5,6]. Additionally, inbred rat strains vary in their susceptibility to carcinogen-induced mammary cancer, allowing for the identification of genetic susceptibility loci through quantitative trait loci (QTL) analysis. This approach was applied in our lab, utilizing the mammary cancer resistant Wistar-Kyoto (WKy) and susceptible Wistar-Furth (WF) inbred rat strains resulting in the identification and subsequent fine-mapping of the Mammary Carcinoma Susceptibility 5c, *Mcs5c*, locus [9–11]. *Mcs5c* maps to a 170kb region located in a large gene desert on rat chromosome *5* that shares homology with mice and humans (Fig 1). In both chemical carcinogen and oncogene-induced models of mammary cancer, congenic lines homozygous for the resistant WKy *Mcs5c* allele showed an approximately 50% reduction in carcinoma number compared to susceptible WF-homozygous controls [11].

Using the *Mcs5c* locus as a model, we sought to examine the interaction between a genetic risk factor and WOS. We have characterized an 8.5kb temporal control element (TCE) within *Mcs5c* affecting the expression of neighboring gene Pregnancy-associated plasma protein A, *Pappa*, in a genotype-dependent manner in mammary epithelial cells (MECs). The function of the Pappa/PAPP-A protein makes it an attractive candidate for involvement in both the WOS phenomenon and breast cancer development. PAPP-A is a protease that acts to positively regulate bioavailability and signaling of the Insulin-like growth factors, IGFs, through the cleavage of IGF binding proteins 2, 4 and 5, IGFBP2/4/5 [12–15]. The specific role of PAPP-A in normal breast development has not been studied, but the IGF-I pathway, in general, is an essential component of breast/mammary gland development, as evident by the severe mammary gland defects of *Igf-I* and *Igf-I* receptor (*Igf1r*) knockout mice [16–18]. The role of IGF-I in breast cancer development is supported by numerous studies which associate the IGF-I signaling pathway with breast cancer initiation and progression [19]. Indeed, in transgenic mice, overexpression of IGF-I in the mammary gland resulted in increased susceptibility and decreased latency to spontaneous and carcinogen-induced mammary adenocarcinomas [20]. Limited studies of PAPP-A function in cancer have demonstrated that increased PAPP-A activity enhanced tumor growth in ovarian and lung cancer cell lines [21,22], and inhibition of its proteolytic function reduced tumor growth in a murine mammary cancer cell line [23]. Furthermore, TCGA data [24] found *PAPP-A* to be altered in 6% of invasive breast carcinomas, with amplification/mRNA upregulation identified as the most common genetic alterations, and found co-amplification of neighboring loci, encompassing the homologous *MCS5C* locus, occurring in approximately 1–2% of cases (accessed via www.cbioportal.org; [25,26]).

In this study, we have identified age-specific differences in *Mcs5c* activity which support the existence of mechanistically distinct susceptibility windows. We have functionally characterized the non-coding *Mcs5c* locus, finding that it acts during the aWOS to regulate *Pappa* expression through age-dependent chromatin looping and genotype-dependent DNA methylation. To our knowledge, this study represents the first identification of a molecular mechanism underlying the aWOS phenomenon and highlights the ability of developmental age to influence the activity of a susceptibility locus.

## Results

### *Mcs5c* acts in a mammary gland autonomous manner

**Fig 2:**
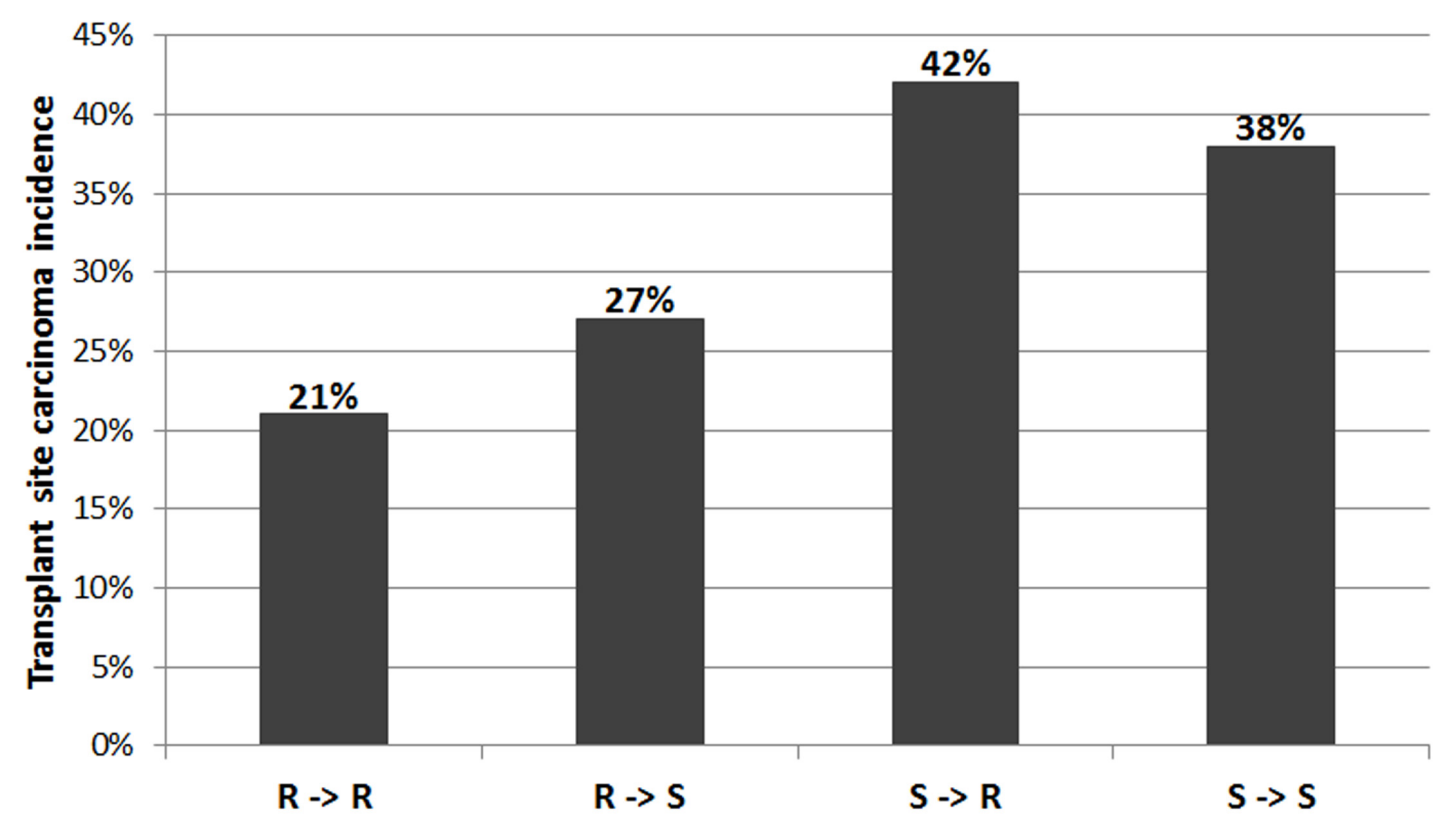
*Mcs5c* acts in a mammary gland autonomous manner to influence carcinoma multiplicity. The four mammary gland transplant groups are listed on the x-axis (R = *Mcs5c* resistant 5C-27 line, S = *Mcs5c* susceptible control line), with the genotype of the donor listed first and the genotype of the recipient listed second. The number of animals per transplant group were: R->R, n = 76; R->S, n = 49; S->R, n = 69; S->S, n = 39. The y-axis indicates the percentage of animals in each group that had one or more carcinomas at the transplant site 15 weeks after DMBA administration. Logistic regression analysis found a statistically significant donor effect (p-value = 0.0043; recipient effect p-value = 0.825).

To determine if *Mcs5c* exerts its effect on carcinoma multiplicity via the mammary gland, transplant experiments were performed. Donor mammary gland tissue from either the *Mcs5c* resistant 5C-27 line or a *Mcs5c* susceptible control line was transplanted onto the interscapular fat pad of recipient rats from both genotypes, creating four donor-recipient groups. This direct transplant design allowed for the detection of mammary gland-host interactions and did not result in differential tissue rejection rates, as the lines are isogenenic except at the *Mcs5c* locus. Transplant tissue rejection rates were not statistically significant between transplant groups consisting of donors and recipients with the same genotype versus groups with different genotypes (Chi-squared test, X = .10, df = 1, p-value = 0.75). Results from the mammary gland transplant experiment are shown in Fig 2. Resistant and susceptible rats receiving resistant donor tissue had a transplant site carcinoma incidence of 21% and 27%, respectively (n = 76, 49), while resistant and susceptible rats receiving susceptible tissue had incidences of 42% and 38%, respectively (n = 69, 39). Recipient rats of either genotype that received susceptible donor tissue had higher transplant site carcinoma incidences than those that received tissue from resistant rats. In this way, the carcinoma phenotype was dependent on the donor tissue genotype and was not influenced by the recipient’s genotype, suggesting that *Mcs5c* acts within the mammary gland. Indeed, logistic regression analysis found a statistically significant donor effect (p-value = 0.0043; recipient effect p-value = 0.825). Thus, it was concluded that *Mcs5c* acts in a mammary gland autonomous manner to influence carcinoma multiplicity.

### *Pappa* is differentially expressed in MECs in an age-dependent manner

**Fig 3:**
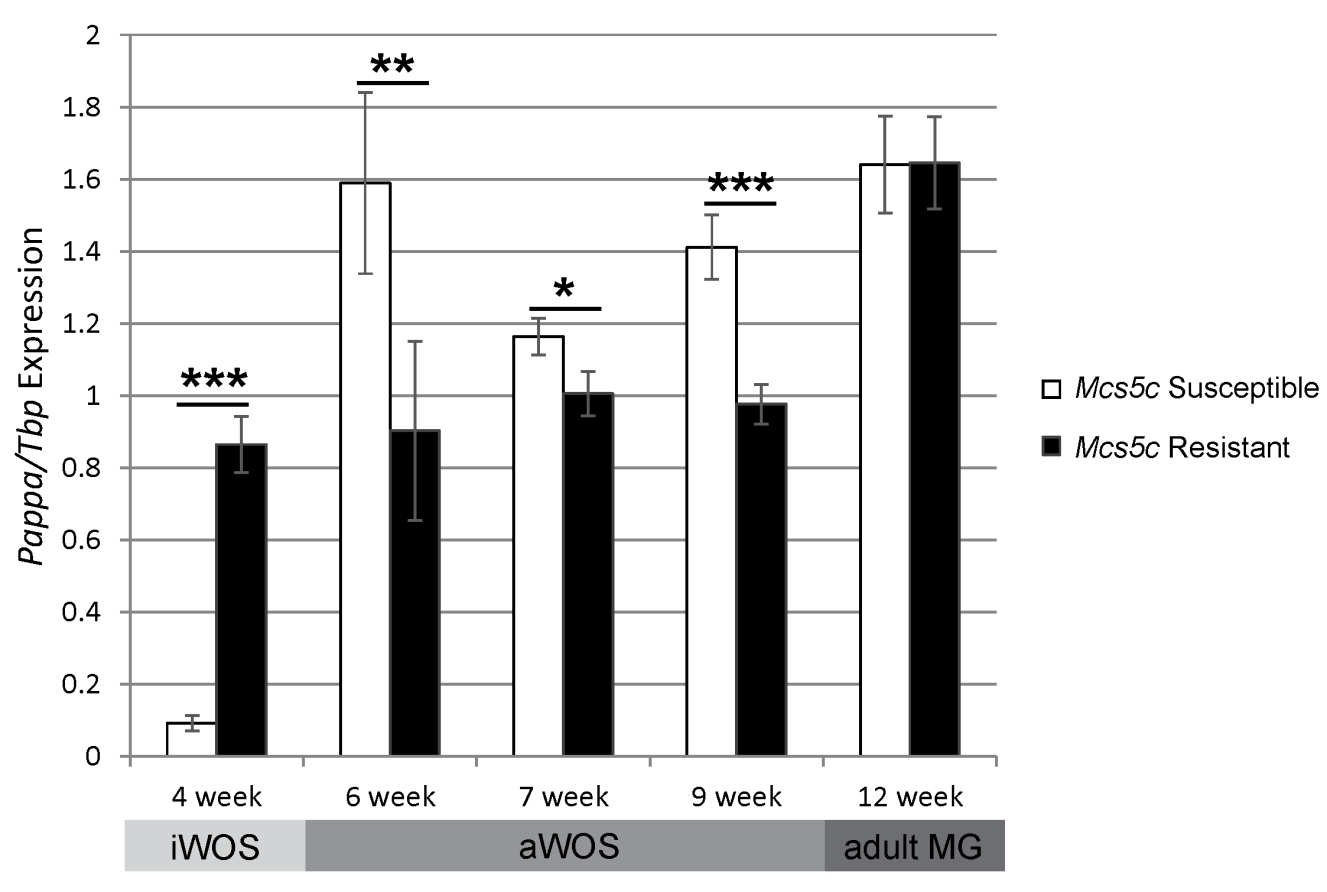
*Pappa* expression is altered in *Mcs5c* susceptible rats in an age-dependent manner. *Pappa* expression was examined in MECs of *Mcs5c* susceptible and *Mcs5c* resistant rats at ages falling within the iWOS, aWOS, and adult mammary gland, as indicated below the graph. Relative gene expression was determined via quantitative real-time PCR and standardized to *Tbp* expression. P-values were obtained using the non-parametric Mann-Whitney U test and standard error bars are shown (MG–mammary gland; *, P ≤ 0.05; **, P ≤ 0.01; ***, P ≤ 0.001).

Quantitative real-time PCR (qPCR) was used to investigate expression levels of nearby genes in mammary epithelial cells (MECs) of *Mcs5c* resistant and susceptible rats at 4–12 weeks of age. This age range was chosen as it captures multiple mammary gland developmental windows, including the iWOS (4 weeks), aWOS (6–9 weeks), and adult (12 weeks) time periods. *Pappa*, located over 517kb away from *Mcs5c*, was found to be differentially expressed in MECs in an age-dependent manner (Fig 3). In general, *Pappa* expression levels were dynamic in *Mcs5c* susceptible MECs during development, while *Mcs5c* resistant expression remained relatively steady over time. Compared to *Mcs5c* resistant rats, *Pappa* expression was increased in susceptible rats by 43% at 6 weeks (Mann-Whitney U test, p-value = 0.015, n = 13 and 15, respectively), 14% at 7 weeks (p-value = 0.05, n = 23 and 19), and 31% at 9 weeks (p-value = 0.0003, n = 23 and 18). Differential expression disappeared by 12 weeks of age (n = 9 and 18), at which point the mammary gland is fully developed and rats are past the aWOS stage [5]. Expression trends were reversed in 4 week old rats, with susceptible animals showing a sharp decrease in expression relative to resistant rats (p-value = 8e-5, n = 9 and 8, respectively). *Mcs5c*, therefore, appears to functioning during both the iWOS and aWOS. Unfortunately, we were unable to obtain robust antibodies for analysis of Pappa protein levels in mammary gland tissue. Differential expression in MECs was not observed for neighboring genes Tenascin C, *Tnc*, and Tumor Necrosis Factor (Ligand) Superfamily, Member 15, *Tnfsf15*, during the aWOS. However, differential expression of *Tnfsf15* was observed in 4 week-old, immature MECs, highlighting the complexity and age-specific nature of *Mcs5c* locus activity (S1 Fig).

### *Mcs5c* and *Pappa* physically interact in an age-dependent and tissue-specific manner

**Fig 4:**
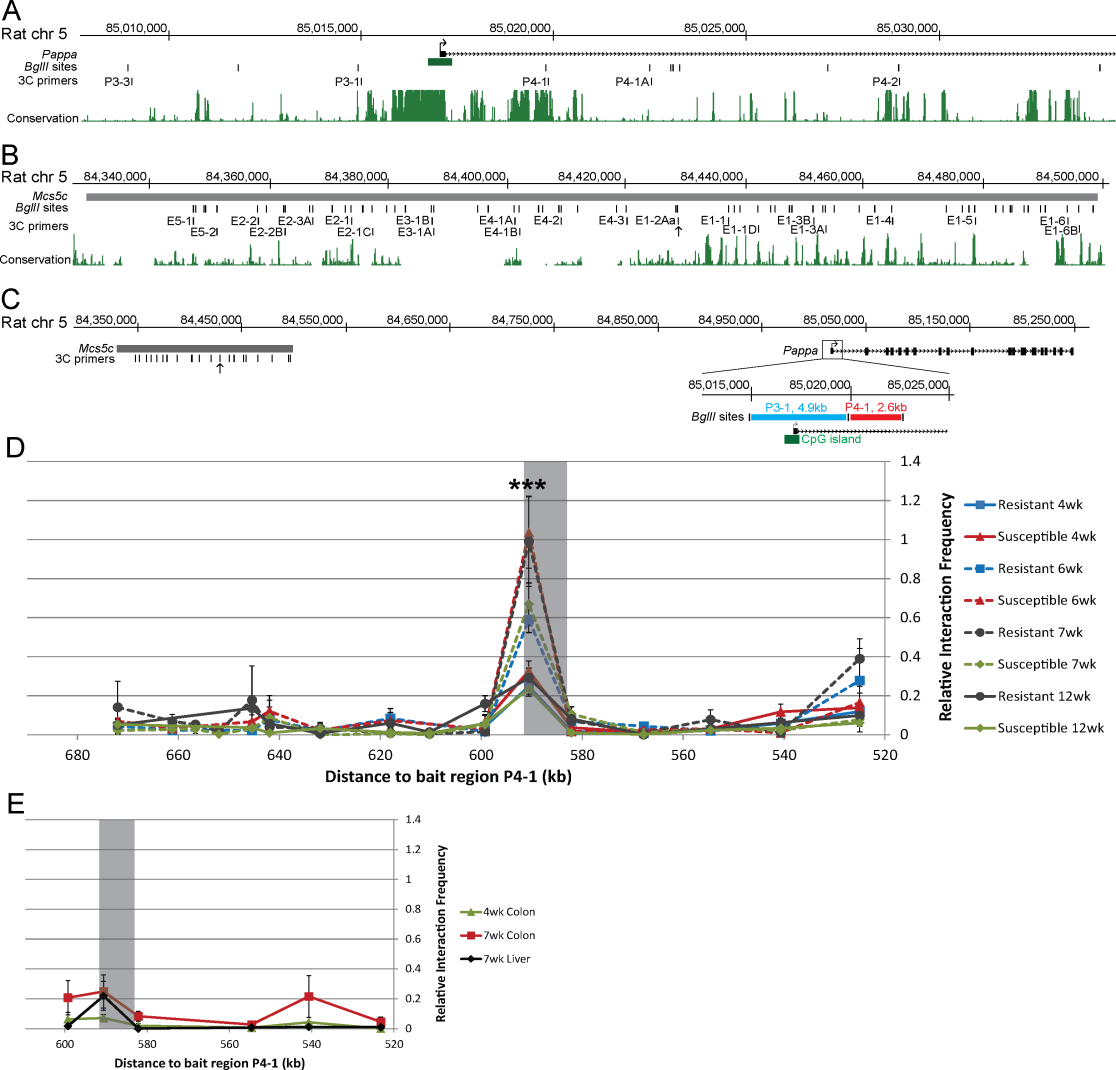
*Mcs5c* displays an age-dependent and tissue-specific interaction with the *Pappa* gene in MECs. A section of the *Pappa* gene (A) and the full-length *Mcs5c* locus (B) are shown with *BglII* restriction enzyme sites and corresponding primers used for 3C analysis. The TCE is marked by 3C primer E1-2Aa (arrow). Vertebrate conservation from the UCSC Genome Browser (March 2012, rn5) PhastCons track is also shown. (C) The *Mcs5c* locus is shown in relation to the full length *Pappa* gene, with bait regions P3-1 (blue line) and P4-1 (red line) highlighted in the zoomed in image. *BglII* sites and 3C primers are indicated, with an arrow marking the TCE. The green box highlights a conserved CpG island. (D) MEC 3C profile showing the relative interaction frequency (IF, y-axis) between the P4-1 bait region and regions spanning the entire *Mcs5c* locus. The x-axis indicates the distance between the tested region in *Mcs5c* and the P4-1 bait region. The shaded box highlights the temporal control element (TCE) within *Mcs5c* that displayed an age-specific interaction with P4-1, with 6 and 7 week samples showing a strong interaction (dotted lines) and 4 and 12 week samples a negative/weak interaction (solid lines). (E) Colon epithelial cell and liver hepatocyte 3C profiles showed a negative/weak interaction between the TCE (shaded box) and bait region P4-1. The x-axis is the same as in (D), however, not all sites within *Mcs5c* were tested, resulting in a truncated axis. Only *Mcs5c* resistant rats were used for this analysis. For both (D) and (E), each point represents multiple biological and technical replicates, and standard error bars are included. P-values were obtained using the non-parametric Mann-Whitney U test (*, P ≤ 0.05; **, P ≤ 0.01; ***, P ≤ 0.001).

Genotype dependent differential expression seen in MECs led to the hypothesis that *Mcs5c* contained a long-distance acting regulatory element influencing *Pappa* expression. Such a relationship could be mediated by a physical association between the two regions, resulting in the looping out of intervening DNA sequence. Chromosome conformation capture (3C) was used to identify such an interaction. To create 3C templates, MECs were isolated from the mammary glands of *Mcs5c* resistant and susceptible animals at 4, 6, 7, and 12 weeks of age. Two fixed bait regions located at the *Pappa* locus were chosen for extensive analysis of potential interactions with *Mcs5c*. These regions, P3-1 and P4-1, span approximately 2.4kb and 2kb in size, respectively, with P3-1 encompassing *Pappa* exon one and a conserved CpG island, and P4-1 falling within the first intron (Fig 4A and 4C). These two regions were chosen for analysis as their degree of sequence conservation suggested that they may be functionally relevant in transcriptional regulation of the *Pappa* gene (Fig 4A). Bait region P3-1 was negative for any interaction with *Mcs5c* at 4, 7, and 12 weeks of age (S2 Fig). Conversely, 3C analysis using bait region P4-1 revealed an 8.5kb region within *Mcs5c* that displayed a high relative interaction frequency (IF) in 6 and 7 week templates, indicative of a physical interaction between the two regions occurring over a distance of 590kb (Fig 4D). 4 and 12 week templates had a much lower IF at this -590 region, leading to the formation of two distinct, age-dependent interaction groups displaying either a strong (6 and 7 week) or weak (4 and 12 week) IF. The difference in IF for these two groups was statistically significant (Mann-Whitney U test, p-value = 1.02e-10, n = 27 and 38 biological replicates, respectively). For all ages, there was no difference in IF between genotypes, indicating that the interaction is age-dependent but not genotype-dependent. We will therefore refer to the -590 looping region of *Mcs5c* as the temporal control element (TCE; chr5:84,428,694–84,437,192; RGSC 5.0/rn5). Three additional *Pappa* bait regions were tested for interactions with the *Mcs5c* TCE at 4 and 6 weeks of age (S2 Fig). Two of these regions, P4-1A and P4-2, were negative, while the more proximal P3-3 region displayed an aWOS-specific looping interaction that mimicked the TCE/P4-1 interaction. This indicates that the *Mcs5c* TCE may utilize a more complex looping scheme to facilitate *Pappa* regulation, and defines the TCE as a functionally important region within *Mcs5c*. To determine if these interactions are also tissue-specific, 3C profiles were analyzed from 4 and 7 week colon epithelial cells and 7 week liver hepatocytes from *Mcs5c* resistant rats. The *Mcs5c* TCE did not interact with P4-1 (Fig 4E) or P3-3 (S2 Fig) in these tissues, implying that the interactions between *Pappa* and the *Mcs5c* TCE are tissue-specific in addition to age-dependent. Sequencing of the resistant WKy and susceptible WF TCE alleles revealed 10 variants between the two (S7 Table), and although our 3C results showed that age-specific looping occurs independent of genotype, we speculate that one or more variants may be involved in genotype-dependent expression differences observed during this time period.

### The *Pappa* CpG island shore is differentially methylated *in vivo* in a genotype-dependent manner

**Fig 5:**
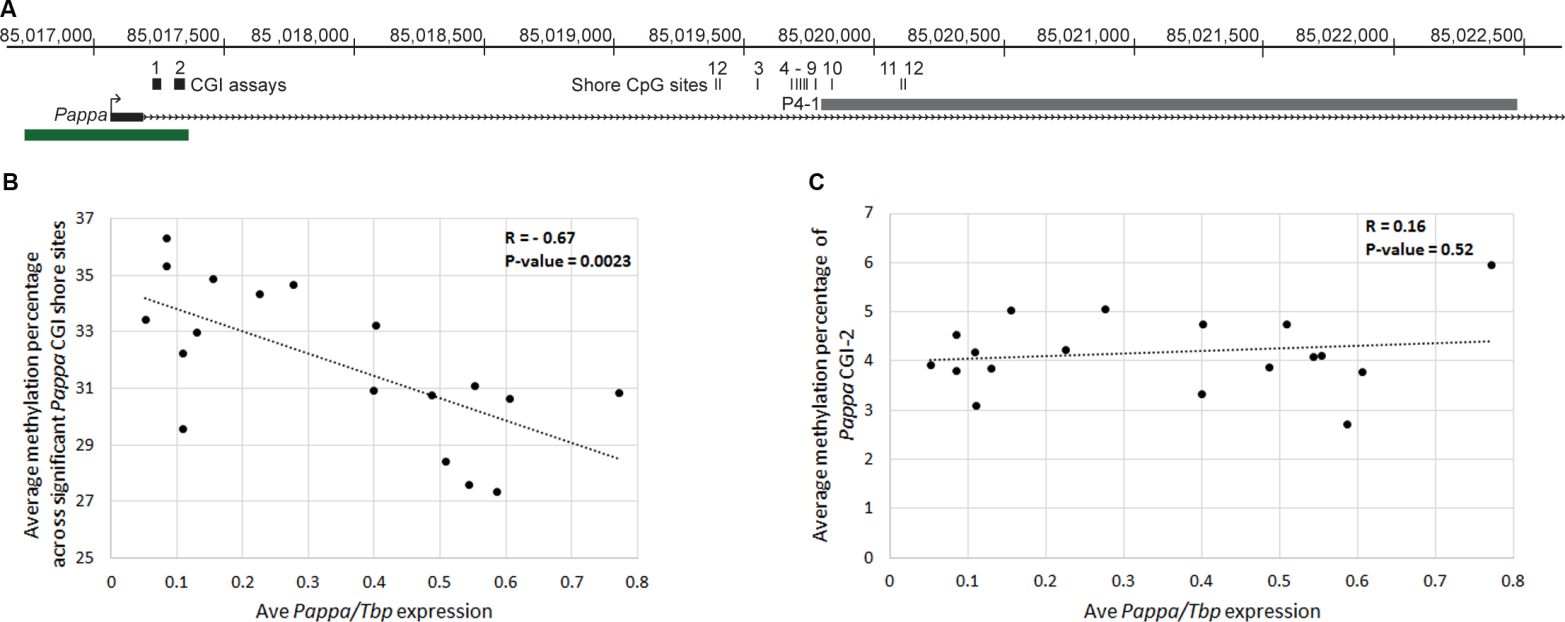
*In vivo* methylation analysis of the *Pappa* CGI and CGI shore. (A) The first exon of the *Pappa* gene is shown in relation to a conserved CGI (green box) and the P4-1 looping fragment (gray box). The location of the 12 shore CG dinucleotides investigated in this report are indicated and numbered, as are the regions covered by the two pre-made CGI pyrosequencing assays. The CGI assays each examined 5 CG dinucleotides within the island. (B) A scatterplot demonstrating a statistically significant negative correlation between 6 week MEC *Pappa* expression (x-axis) and shore methylation (y-axis; Pearson correlation coefficient, R, = -0.67, n = 18, p-value = 0.0023) is shown. Shore methylation values were obtained by averaging the absolute methylation percentages of the 6 significant shore sites (Sites 1, 3, 6–9) for each individual sample. A linear trend line is shown with the dotted line (slope = -7.88). (C) No correlation was observed between 6 week MEC *Pappa* expression (x-axis) and CGI methylation (y-axis; Pearson correlation coefficient, R, = 0.16, n = 18, p-value = 0.52). CGI methylation values were obtained by averaging the absolute methylation percentages of the 5 sites examined by the CGI-2 assay for each individual sample. A linear trend line is shown with the dotted line (slope = 0.544).

CpG island (CGI) shores are regions located approximately 2kb away from CGIs, and have increasingly been identified as the sites of tissue specific differential methylation associated with gene expression changes [27]. The *Pappa* looping fragment, P4-1, resides in a CGI shore region (Fig 5A). As this region is a target site of *Mcs5c* TCE looping, we hypothesized that *Mcs5c* may affect *Pappa* expression through an epigenetic mechanism targeted to the P4-1 fragment. Methylation levels for 12 CG dinucleotides within and proximal to P4-1 were examined in MECs of *Mcs5c* resistant and susceptible rats at 4, 6, 7, 9 and 12 weeks of age using custom designed pyrosequencing assays (Fig 5A). Selection of these timepoints allowed for the examination of methylation patterns before, during, and after the aWOS. In general, methylation levels were dynamic across this region, with sites 2–4 consistently displaying the lowest methylation levels (average = 13% methylated) and sites 9–12 displaying the highest levels (average = 68% methylated) (S4 Table). Additionally, there appeared to be few age-specific differences in methylation levels for animals within the aWOS, therefore data for 6, 7, and 9 week old rats were combined within genotypes. Of the 12 sites examined, 6 showed statistically significant genotype-dependent differences in methylation levels after adjusting for multiple comparisons (Mann-Whitney U-test with Bonferroni correction). The percent change in methylation levels along with p-values are shown in Table 1. All statistically significant, genotype-dependent methylation differences occurred during the aWOS and were directionally identical, with methylation levels decreased in *Mcs5c* susceptible MECs. The percent decrease in methylation levels ranged from 5.0%– 22.7%. Additionally, a number of other sites displayed a similar trend, although these differences were not significant after Bonferroni correction. At ages outside of the aWOS, there were no statistically significant genotype-dependent differences in methylation, although sites 1 and 2 displayed a non-significant trend of increased methylation in *Mcs5c* susceptible MECs at the 4 week time point. We also investigated the methylation state of the *Pappa* CGI using 2 pre-made pyrosequencing assays (Fig 5A). Methylation levels for both assays were assessed in 4 week old animals, while one assay was examined at the remaining timepoints. For all CGI assays and timepoints, there were no genotype-dependent differences in methylation levels and, in general, the *Pappa* CGI is hypomethylated at all ages, with site specific methylation levels ranging from 0.16% - 8.41% (S5 Table). The observation of decreased shore methylation and increased *Pappa* expression in *Mcs5c* susceptible MECs strongly supports the canonical role of DNA methylation in gene regulation, that is, that the two are negatively correlated. Indeed, for 6 week MECs, for which we had both DNA and RNA samples, *Pappa* expression was negatively correlated with the average methylation percentage of the 6 significant shore sites (Fig 5B; Pearson correlation coefficient, R, = -0.67, n = 18, p-value = 0.0023). By contrast, no correlation was observed between *Pappa* expression and the average methylation percentage of the CGI-2 assay sites (Fig 5C; Pearson correlation coefficient, R, = 0.16, n = 18, p-value = 0.52). The identification of genotype-dependent methylation differences during the aWOS suggests that *Mcs5c* facilitates genotype-dependent *Pappa* expression differences observed during this time period through epigenetic modification of the *Pappa* CGI shore.

**Table 1:** Percent change in *Mcs5c* susceptible MEC methylation at sites within the *Pappa* CGI shore relative to *Mcs5c* resistant MECs.

	Site 1	Site 2	Site 3	Site 4	Site 5	Site 6	Site 7	Site 8	Site 9	Site 10	Site 11	Site 12
**4 weeks (iWOS)**	+22.6 (NS)	+26.1 (NS)	(NS)	(NS)	(NS)	(NS)	(NS)	(NS)	(NS)	(NS)	(NS)	(NS)
**6/7/9 weeks (aWOS)**	-20.4 (0.0002)	-23.1 (NS)	-22.7 (0.0004)	-12.6 (NS)	-5.1 (NS)	-12.2 (0.004)	-13.4 (0.003)	-8.9 (0.0114)	-5.0 (0.023)	-5.6 (NS)	-2.2 (NS)	(NS)
**12 weeks (adult MG)**	(NS)	(NS)	(NS)	(NS)	(NS)	(NS)	(NS)	(NS)	(NS)	(NS)	(NS)	(NS)

The direction of the percent change in *Mcs5c* susceptible MEC methylation levels relative to *Mcs5c* resistant MEC levels is indicated by a + (increase) or—(decrease). Bonferroni corrected p-values are shown in parentheses and non-significant percent change trends are also shown (Mann-Whitney U Test; MG–mammary gland; NS–not significant).

### Removal of the *Mcs5c* TCE decreases *Pappa* expression and increases shore methylation *in vitro*

**Fig 6:**
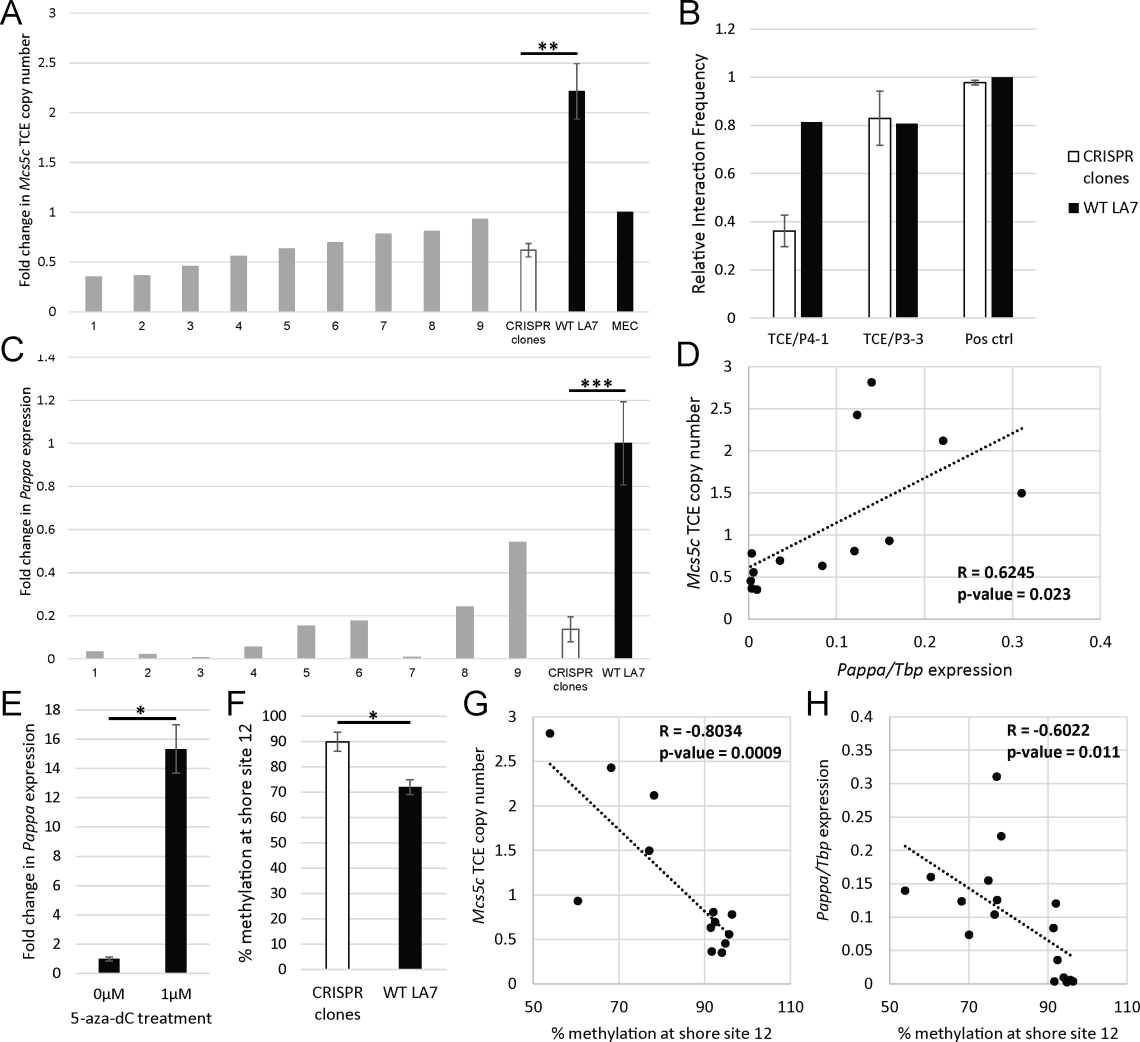
Removal of *Mcs5c* TCE copies results in decreased *Pappa* expression *in vitro*. (A) CRISPR guides targeting the TCE were transfected into LA7 cells, and clones were screened for removal of the target region. Nine positive clones were assessed for remaining TCE copy number via qPCR, standardized to a non-targeted region within the *Pappa* gene. Copy number in wild-type LA7 cells (n = 4 independent cultures) was also assessed, and results were normalized to diploid MECs. (B) Interaction frequency (IF, y-axis) was calculated in select positive clones (n = 3) and WT LA7 cells between the TCE and *Pappa* bait regions P4-1 and P3-3. The IF for a positive control region, two nearby *BglII* fragments, is shown for reference. (C) *Pappa* expression in positive clones and WT LA7 cells (n = 6) was analyzed via qPCR and standardized to *Tbp* expression. (D) A scatterplot of *Pappa* expression and *Mcs5c* TCE copy number demonstrate a statistically significant positive correlation between the two (Pearson correlation coefficient, R, = 0.6245, n = 13, p-value = 0.0225). A linear trend line is shown (slope = 5.327). (E) WT LA7 cells were treated with 0μM (n = 4) or 1μM (n = 4) 5-aza-dC for 48hrs. *Pappa* expression was analyzed via qPCR and standardized to *Tbp*. (F) Methylation levels at *Pappa* CGI shore site 12 are shown for WT LA7 cells (n = 8) and CRISPR clones (n = 9). The p-value reflects Bonferroni correction. Scatterplots demonstrating negative correlations for shore site 12 methylation levels and *Mcs5c* TCE copy number (G) and *Pappa* expression (H) are shown (R = -0.8034/-0.6022, n = 13/17, p-value = 0.0009/0.011, respectively) along with linear tread lines (slope = -0.046/-0.004, respectively). For all bar graphs, p-values were obtained using the non-parametric Mann-Whitney U test, and standard error bars are shown (*, P ≤ 0.05; **, P ≤ 0.01; ***, P ≤ 0.001).

In an effort to causally tie the *Mcs5c* TCE to *Pappa* expression and CGI shore methylation, the entire 8.5kb region was targeted for deletion in the rat mammary carcinoma cell line, LA7. Two CRISPR guides were used to target the region, and clones were screened via PCR across the cut site, with validation by sequencing (S1 Table). We were unable to identify a clone with all copies of the TCE removed, despite much effort. This was likely due to the aneuploid nature of LA7 cells, and mutations incurred at CRISPR guide target regions (S2 & S3 Tables). Copy number analysis of 9 positive CRISPR edited clones showed that we were able to delete a majority of TCE copies, reducing the copy number by 3.5-fold across all clones (Fig 6A). 3C analysis of positive clones indicated that removal of multiple TCE copies resulted in decreased TCE/P4-1 looping, but did not alter TCE/P3-3 looping, which remained consistent with WT levels (Fig 6B). Interestingly, this suggests that the looping mechanisms responsible for these interactions are functionally distinct. Expression analysis revealed a significant reduction in *Pappa* expression in CRISPR clones compared to wild-type LA7 cells, with *Pappa* decreased 4-fold across all clones (Fig 6C). A Pearson correlation coefficient was computed to determine the relationship between *Pappa* expression and TCE copy number, and a positive correlation between the two was observed (Fig 6D; R = 0.6245, n = 13, p-value = 0.0225). Conversely, no change in *Tnc* or *Tnfsf15* expression was observed with TCE knockdown, and expression levels were not correlated to TCE copy number (S3 Fig). These data support our hypothesis that *Mcs5c* contains a long-range regulatory element, and emphasizes the functionality of the TCE/P4-1 chromatin loop to *Pappa* gene expression.

Our *in vivo* analysis highlighted the importance of *Pappa* CGI shore methylation to *Pappa* expression, and we sought to verify this relationship in our *in vitro* model as well. Treatment of wild-type LA7 cells with the DNA methylation inhibitor 5-aza-2’-deoxycytidine (5-aza-dC) resulted in a 15-fold increase in *Pappa* expression (Fig 6E), indicating that DNA methylation plays a role in *Pappa* regulation. To more specifically address the relationship between TCE/P4-1 looping and *Pappa* methylation, CGI and CGI shore methylation levels were analyzed in wild-type LA7 cells and CRISPR edited clones (S10 Table). Two CGI shore sites showed statistically significant differences in methylation, with a 3.9% decrease and a 25% increase in methylation levels observed in CRISPR clones at sites 5 site 12, respectively (S10 Table and Fig 6F). Methylation changes at site 12 were more pronounced, and were investigated further. We found site 12 methylation levels to negatively correlate with TCE copy number (Fig 6G; R = -0.8034, n = 13, p-value = 0.0009). Site 12 methylation levels were also negatively correlated with *Pappa* expression (Fig 6H; R = -0.6022, n = 17, p-value = 0.011), mimicking the observed *in vivo* relationship. Altogether, these data suggest a functional chain of events whereby the TCE, via the TCE/P4-1 loop, affects *Pappa* CGI shore methylation levels which then, in turn, affect *Pappa* expression.

## Discussion

Previous work on *Mcs5c* had fine-mapped the locus to a 170kb non-coding region on rat chromosome *5*. This locus resulted in an approximately 50% decrease in both chemical carcinogen and oncogene-induced mammary carcinoma development when homozygous for the resistant WKy allele. The gene *Tnc* was initially identified as a possible target of *Mcs5c* activity, with genotype-dependent differential expression observed in the thymus and ovaries exclusively following carcinogen exposure [11]. However, in this study, we have shown that the *Mcs5c* locus affects carcinoma multiplicity in a mammary gland autonomous manner (Fig 2). This suggests that the previous non-mammary gland expression differences observed following carcinogen exposure do not play a role in carcinoma initiation, and are either irrelevant or secondary to initial carcinoma development that is dependent on mammary gland intrinsic factors. While these hypotheses warrant further investigation, a reevaluation of gene expression within the mammary gland was conducted, revealing genotype-dependent and age-specific differential expression of *Pappa* in mammary epithelial cells (MECs; Fig 3). Specifically, *Mcs5c* susceptible MECs from 6 to 9 week old rats showed increased expression of *Pappa* compared to *Mcs5c* resistant rats. Importantly, the 6 to 9 week age range encompasses a time period of rapid mammary gland development and maturity, falling within the aWOS [5].

We hypothesized that *Pappa* expression changes were mediated by a regulatory element within *Mcs5c*, and our experimental results support this hypothesis, identifying a complex set of mechanisms underlying *Mcs5c*-mediated regulation of *Pappa*. Through 3C experiments, we have identified a region within *Mcs5c*, the temporal control element (TCE), that physically interacts with the *Pappa* locus at two regions, P4-1 and P3-3, in an aWOS- and MEC-specific manner over distances of 590kb and 580kb, respectively (Fig 4 and S2 Fig). The importance of the TCE/P4-1 long-range looping interaction to *Pappa* expression was demonstrated *in vitro*, where removal of TCE copies resulted in a reduction of TCE/P4-1, but not TCE/P3-3, looping, and correlated with decreased *Pappa* expression (Fig 6). These data indicate that the two observed TCE chromatin interactions are functionally distinct, and demonstrates a strong positive regulatory relationship between the *Mcs5c* TCE and *Pappa* expression, which appears to be dependent on TCE/P4-1 chromatin looping. The *Mcs5c* TCE/*Pappa* P4-1 interaction, therefore, represents another example of a long-distance acting regulatory region, akin to those identified for the *Shh* [28] and *Sox9* [29] genes, as well as the previously characterized *Mcs1a* locus [30].

Importantly, as looping occurs in a genotype-independent manner, additional mechanisms must be responsible for the differential expression observed between *Mcs5c* resistant and susceptible rats. With the intronic P4-1 looping region falling in a CGI shore, DNA methylation of this region became a mechanistic candidate to explain observed expression differences. The importance of differentially methylated CGI shores to gene expression was first highlighted by Irizarry and colleagues in 2009 [27]. Since then, many studies have shown an association between differentially methylated shore regions and gene expression changes [31–38]. Our study identified 6 CG dinucleotides within and proximal to the P4-1 looping region that were differentially methylated between *Mcs5c* resistant and susceptible MECs (Table 1). Significant methylation differences were observed exclusively during the aWOS, and a negative correlation between shore methylation levels and *Pappa* expression strongly suggest that DNA methylation plays a role in differential *Pappa* expression (Fig 5B). This correlation was recapitulated in our *in vitro* model, where differential shore methylation was also negatively correlated with TCE copy number (Fig 6G and 6H). As copy number acts as an indicator of TCE/P4-1 looping frequency in this model, this suggests a functional relationship between looping and shore methylation, where the TCE/P4-1 loop acts to facilitate differential methylation that, in turn, regulates *Pappa* expression. Given the inherent difficulties of modeling an age-dependent phenomenon *in vitro*, these results must be interpreted cautiously; however, we feel that the similarities between our *in vitro* and *in vivo* results indicate that these mechanisms are robust and functionally relevant to *Mcs5c*-mediated *Pappa* regulation.

Overall, we have identified two mechanisms associated with *Mcs5c* regulation of *Pappa* expression during the aWOS, chromatin looping and DNA methylation. Our *in vitro* experiments have indicated the importance of the TCE/P4-1 loop for *Pappa* expression and shore methylation; however, *in vivo* analyses have shown that these actions are mechanistically distinct, as *Pappa* expression and differential methylation, but not looping, are genotype-dependent. An unresolved issue is precisely how *Mcs5c* is mediating these activities. We hypothesize that the genotype-independent TCE/P4-1 loop serves to facilitate the recruitment of transcription factors, cofactors, and/or methyltransferases that act separately or together to directly regulate *Pappa* methylation and expression during the aWOS. The binding of these regulatory factors would be affected by one or more variants within the TCE without affecting chromatin looping. Wright *et al*. [39] identified a similar interaction at the c-*MYC* locus, where an enhancer-associated SNP affected transcription factor binding without altering chromatin structure. Sequencing of the resistant WKy and susceptible WF *Mcs5c* TCE alleles (chr5:84,428,694–84,437,192; RGSC 5.0/rn5) has revealed 10 candidate polymorphisms (S7 Table) for future investigation of their effect on protein binding and subsequent *Pappa* expression and methylation changes.

*Mcs5c* activity during the aWOS stands in stark contrast to that observed during the iWOS (4 weeks). Specifically, differential *Pappa* expression during the iWOS is reversed compared to the aWOS (Fig 2), TCE/*Pappa* looping is lacking (Fig 4D and S2 Fig), and there are no statistically significant CGI shore methylation differences (Table 1). These data indicate that the regulatory actions of *Mcs5c* are dependent on developmental context, a phenomenon observed at other regulatory regions, most notably the β-globin locus control region [40]. Age-specific differences in *Pappa* expression, looping, and methylation could be explained by interactions with proteins specific to these developmental time points. Identifying proteomic differences between the immature and adolescent mammary gland will be crucial in understanding the players driving window-specific mechanistic differences in *Mcs5c* activity. We hypothesize that age-specific protein expression results in an alternative looping interaction between *Mcs5c* and *Pappa* during the iWOS. Differential expression of *Tnfsf15* exclusively at the 4 week time point (S1 Fig) indicates that *Mcs5c* may exhibit a more complex chromatin interaction during the iWOS, regulating multiple genes simultaneously. Additionally, a trend towards increased methylation in *Mcs5c* susceptible MECs is functionally consistent with the reduction of *Pappa* expression observed during this time period. It is possible that these sites are indicative of significant methylation differences occurring at sites not examined in this study, both at the *Pappa* locus as well as *Tnfsf15*, and shore methylation may still, therefore, be relevant to *Mcs5c* activity during the iWOS.

**Fig 7:**
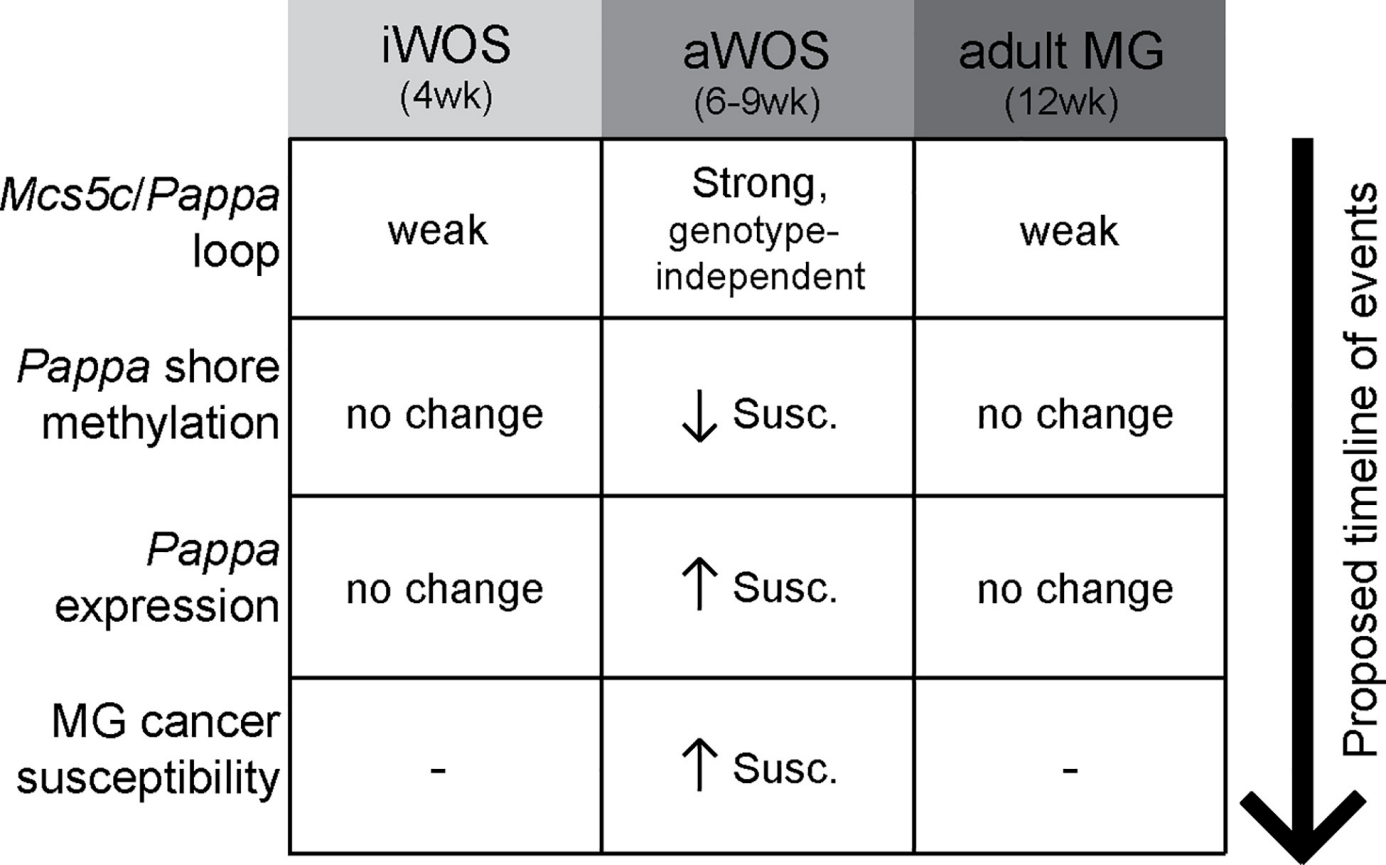
Summary of *Mcs5c* activity within MECs. Our experimental finding are summarized according to the specific time periods examined in this study. The aWOS is the time period during which *Mcs5c* is most active in MECs, functioning in a genotype-independent manner with regards to looping, and a genotype-dependent manner with regards to *Pappa* CGI shore methylation and gene expression. *In vivo* and *in vitro* analyses have led to the proposed timeline of events, whereby *Mcs5c* looping results in differential methylation which subsequently affects gene expression. Ultimately, this leads to the previously reported *Mcs5c* genotype-dependent differences in MG cancer susceptibility [11] (MG–mammary gland; Susc.–*Mcs5c* susceptible rats; directionality of changes indicated with small arrows).

Altogether, we have functionally characterized the *Mcs5c* locus, finding that it acts via two distinct mechanisms to influence *Pappa* expression in an age-dependent manner during a well-characterized breast cancer WOS (Fig 7). This work highlights the importance of characterizing genetic risk factors in the context of developmental windows of susceptibility (G x WOS), and emphasizes the complex interaction between genetic, environmental, and age-specific risk factors. *Mcs5c* susceptible rats showed increased expression of *Pappa* in MECs and an increased susceptibility to carcinogen-induced mammary carcinogenesis, supporting a protective benefit of reduced *Pappa* levels during adolescent development. Decreased levels of Pappa in the developing mammary gland would result in reduced Igf-I bioavailability through a reduction in Igfbp cleavage [12]. Given that the Igf-I signaling pathway acts to promote proliferation and inhibit apoptosis during mammary gland development [41], it is therefore likely that a reduction in free Igf-I would reduce the proliferative index of MECs. As the effects of many environmental mutagens, such as radiation and chemical carcinogens, are dependent on interactions with the DNA of proliferating cells [42], this would result in fewer targets for mutagenesis, and represents one possible method by which reduced *Pappa* expression during the aWOS may result in a mammary carcinoma resistant phenotype. Understanding the mechanisms behind G x WOS interactions and how environmental risk factors influence these interactions will play a crucial role in breast cancer risk assessment, and in the identification of targets and strategies for cancer prevention in young women. There is growing concern over the impact adolescent exposure to a broad range of environmental factors may have on long-term breast cancer risk [43]. Our work has demonstrated a functional relationship between genetic risk factor activity and developmental stage, and it is likely that environmental risk factors may further confound such interactions. Indeed, CGI shore methylation has been found to be affected by environmental factors such as ELS and diet [32,33,44,45]. We believe that the *Mcs5c* locus will serve as a robust model to study how environmental factors affect breast cancer risk by influencing G x WOS interactions, and may encourage the characterization of other such cancer susceptibility loci in this context.

## Materials and Methods

### Animals

Congenic rat lines were maintained in an AAALAC-accredited facility as previously described [11]. All protocols were approved by the University of Wisconsin–Madison School of Medicine and Public Health Animal Care and Use Committee. Congenic rat lines are defined as having the resistant Wistar-Kyoto (WKy) *Mcs5c* allele introgressed on a susceptible Wistar-Furth (WF) background. The resistant congenic line used in this study, 5C-27, is WKy-homozygous for a genomic region that includes the entirety of the *Mcs5c* locus (Fig 1) [11]. Susceptible control animals are WF-homozygous at the *Mcs5c* locus.

### Mammary gland transplantation

*Mcs5c* WKy-homozygous congenic rats from line 5C-27 were used as resistant donors and recipients (*Mcs5c* resistant), and *Mcs5c* WF-homozygous rats were used as susceptible controls (*Mcs5c* susceptible). Abdominal and inguinal mammary glands were collected from female donor rats aged 30–35 days old, scissor minced, and split into four equal volumes. One volume was then grafted onto the interscapular white fat pad of four different 30–35 day old female recipient rats. Three weeks after transplantation (51–56 days), recipients were administered the chemical carcinogen 7,12-dimethylbenz(*a*)anthracene (DMBA), as a single oral dose dissolved in sesame oil at 65 mg/kg of body weight to induce mammary carcinoma formation. At 15 weeks post-DMBA, animals were removed from the study and carcinomas present at the transplant site greater than 3x3mm were counted. Generally, recipient rats developed ≤1 carcinoma at the transplant site, so incidence values were used as opposed to multiplicity. Fat pads were whole mounted and stained with aluminum carmine to verify transplant mammary gland growth. Four transplant groups were studied, with resistant 5C-27 or susceptible donor glands transplanted into both resistant and susceptible recipients (R->R, R->S, S->R, S->S). The tissue rejection rate for transplant groups consisting of donors and recipients with the same genotype (R->R, S->S) was compared to the rejection rate for groups with differing donor and recipient genotypes (R->S, S->R) via a Chi-squared test. The effect of donor and recipient genotype on carcinoma incidence, converted to a binary response value, was analyzed using logistic regression with two independent variables (donor genotype and recipient genotype) and no interaction term.

### Tissue collection and DNA/RNA processing

For all experiments, mammary epithelial cell (MEC) isolation began with fresh mammary glands (abdominal and inguinal, with lymph nodes removed) that were finely minced and digested for 2 hours at 37°C in 10 mL of GIBCO Dulbecco’s modified Eagle’s medium/F12 (DMEM/F12; Life Technologies) containing 0.01 g/mL of type III collagenase (Worthington). Cell pellets were collected by centrifugation and resuspended in 5 mL DMEM/F12. The suspension was loaded onto a 40μm nylon filter to eliminate stromal cells and collect mammary ductal fragments, consisting of an enriched MEC population. DNA was isolated from cells via the DNeasy Blood and Tissue kit (Qiagen). To isolate RNA, cells were homogenized in TRI Reagent (Ambion), followed by RNA extraction using the MagMAX-96 for Microarrays Total RNA kit (Ambion). LA7 cells used for downstream analysis were collected via treatment with 0.25% trypsin/EDTA (Life Technologies). RNA was extracted using the RNeasy Mini Kit (Qiagen) and DNA was extracted using the DNeasy Blood and Tissue kit (Qiagen).

### Quantitative real-time PCR

MECs were collected at 4–12 weeks of age from female *Mcs5c* resistant and susceptible control rats. RNA was isolated as described above. For *in vivo* and *in vitro* expression analysis, cDNA was prepared from 1–2μg total RNA using Superscript II reverse transcriptase (Invitrogen). Gene expression was quantified using pre-designed or custom made TaqMan qPCR assays (*Pappa*, Rn01458295_m1, FAM; *Tbp*, Rn01455646_m1, VIC; *Tnc*, probe-FAM 5’ CGAGAGCTGTGATTAGA 3’, primers 5’ GGCTGTCAGAAGGCCAGATG 3’ and 5’ TGCCATGAAGGGATTTGAAGA 3’; *Tnfsf15*, Rn00595596_m1, FAM) and run on an ABI Prism 7900HT (Applied Biosystems). *Tbp* was chosen as the reference gene as its expression has been found to be relatively stable across a variety of rat tissues and during different stages of the estrous cycle in the mammary gland [46]. cDNA was diluted 1:4 or 1:8 and run using reaction conditions described previously [11]. Transcript quantities were calculated as described in Smits *et al*. [30], using a standard curve method to calculate C_t_ values and extrapolate quantity values. Sample measurements are an average of 3–4 technical replicates and data were analyzed using SDS software version 2.2.2 (Applied Biosystems).

### Chromosome conformation capture (3C) assay

Sample templates were prepared from MECs, colon epithelial cells, liver hepatocytes, and LA7 cells. MECs were isolated from 4, 6, 7, and 12 week old *Mcs5c* resistant and susceptible rats and the resulting cell suspension was diluted in PBS and fixed via the addition of 1.5% formaldehyde. Colon epithelial cells were isolated from 4 and 7 week old resistant rats, processed as described in Whitehead *et al*. [47], and fixed in formaldehyde. To isolate hepatocytes, the livers of 7 week old resistant rats were digested via cannulation of the portal vein and blanching of the liver using a pre-warmed solution of HBSS (Gibco) + 0.5mM EGTA followed by digestion via pre-warmed DMEM-low glucose (Gibco) + 1000CDU/mL Collagenase type IV (Worthington). Digested livers were collected in DMEM/F12 + 10% FBS on ice and cells dispersed manually. The suspension was filtered through a 100μm nylon filter and the filtrate spun for 2 minutes at 50xg. Supernatant was removed and cell pellets were washed until media became clear, followed by fixation in formaldehyde. Bacterial artificial chromosomes (BACs) encompassing the rat *Mcs5c* and *Pappa* promoter regions (CH230-433D12, CH230-498D4, CH230-256M9, and CH230-244C7) were ordered from Children’s Hospital Oakland Research Institute (CHORI) and used as positive control templates. Subsequent template preparation for all cell types and for BAC controls continued as described in detail in Smits *et al*. [48]. The restriction enzyme used was *BglII*. Chromatin interactions were detected via PCR, with bait primers located at the *Pappa* gene tested against *Mcs5c* primers spanning the entire locus (Fig 4A and 4B). Primer sequences are listed in S6 Table. Reaction components were 1X Herculase reaction buffer, 0.2mM dNTPs, 0.4μM primers, 0.3μμL Herculase Enhanced polymerase (5U/μL, Agilent) in a total volume of 25μμL. The amount of DNA template to add and optimal annealing temperatures were determined empirically. PCR reactions were performed using the following cycling conditions: 95°C for 1 min, 36 cycles of 95°C for 30 s, T_a_ for 30 s, 72°C for 20 s, followed by a final extension of 72°C for 8 min. Reactions were analyzed by agarose gel electrophoresis and visualized by ethidium bromide staining. Band intensities were quantified using ImageQuant software (GE Healthcare). A relative interaction frequency (IF) was calculated by dividing the band intensity of the sample templates by that of the BAC control.

### Sequencing

Sequencing of the 8.5kb *Mcs5c* looping region (TCE; chr5:84,428,694–84,437,192; RGSC 5.0/rn5) identified in 3C experiments was performed on MEC DNA from *Mcs5c* resistant and susceptible rats to assess polymorphisms between the WKy and WF alleles. Sequencing primers are listed in S7 Table. Traditional Sanger sequencing was performed at the University of Wisconsin–Madison Biotechnology Center DNA sequencing facility as described in Smits *et al*. [30].

### Cell culture and *in vitro* CRISPR editing

The rat mammary carcinoma cell line, LA7, was obtained from the American Type Culture Collection and maintained in DMEM/F12 supplemented with 100 IU/mL penicillin, 100 μg/mL streptomycin (Life Technologies), 5% FBS (HyClone), and 0.005mg/mL insulin. Gene expression analysis proceeded as described above, and copy number analysis was performed via SYBR Green qPCR (Life Technologies). For 5-aza-2’-deoxycytidine (5-aza-dC; Sigma) experiments, cells were treated with 1μM 5-aza-dC for 48hrs followed by cell collection and processing. For quantification of *Mcs5c* TCE copies, a primer set located within the CRISPR targeted region was used (5’ CAATCACGTTCACTGTGGGT 3’ and 5’ TCACCTCACACTACCCCATG 3’) and as a control region, a primer set located within the non-targeted *Pappa* gene was used (5’ TCCTCCTGACCACTCTGAGA 3’ and 5’ CCCTACAAACAGCAGAGGGA 3’). The CRISPR-*Cas9* plasmid pSpCas9(BB)-2A-Puro (PX459) was provided by Dr. Feng Zhang (Addgene plasmid #48139) [49]. Guide sequences were designed using the CRISPR Design Tool (http://crispr.mit.edu) and phosphorylated and annealed guide oligos were inserted into the PX459 plasmid via a combination digestion/ligation reaction. 100ng PX459 plasmid was mixed with 2μL of oligos (diluted 1:250), 1μL Fast Digest *BbsI* (Thermo Scientific), 1X Fast Digest Buffer, 1mM DTT, 1mM ATP, and 1500 units T7 ligase (New England BioLabs) and incubated in a thermocycler for 5 minutes at 37°C followed by 5 minutes at 23°C for a total of 6 cycles. The resulting reaction was then treated with Exonuclease V (NEB) according to the manufacturer’s protocol. The product was transformed into competent cells, and colonies expanded and verified via sequencing of the guide insertion site. LA7 cells were transfected with two CRISPR guides flanking the 8.5kb target region. Transfection was performed via electroporation using a Nucleofector II Device and Amaxa Cell Line Nucleofector Kit V (Lonza), according to the manufacturer’s instructions. Stable clones were isolated following puromycin selection, and clonal colonies were expanded. Removal of the targeted region was determined via PCR screening and sequencing. Primers used to create guides and screen clones are listed in S8 Table.

### DNA methylation analysis of the *Pappa* gene

DNA was isolated from wild-type LA7 and CRISPR edited cells as well as *Mcs5c* resistant and susceptible MECs at 4, 6, 7, 9, and 12 weeks of age. Bisulfite conversion was carried out on 500ng of DNA using the EZ DNA Methylation-Lightening kit (Zymo Research), according to the manufacturer’s instructions. Four primer sets were designed to amplify the 12 CpG sites of interest within the *Pappa* CpG island (CGI) shore. Their sequences, along with the sequencing primers used for pyrosequencing, are listed in S9 Table. Optimal amounts of template DNA, MgCl_2_, primers, and annealing temperature were experimentally determined (S9 Table). In general, PCR reactions were performed using the following cycling conditions: 95°C for 5 min, 50 cycles of 95°C for 15 s, T_a_ for 30 s, 72°C for 30 s, followed by a final extension of 72°C for 5 min. 15μL of PCR product was used for pyrosequencing on a PyroMark MD instrument (Qiagen), with 2–3 technical replicates per sample. Data were analyzed using PyroMark CpG software (v 1.0; Qiagen). For analysis of the *Pappa* CGI, 2 pre-made assays were obtained from Qiagen (CGI-1, Rn_D3ZNQ7_01_PM; CGI-2, Rn_D3ZNQ7_02_PM), with PCR conditions following manufacturer’s recommendations. Both pre-made assay CGI-1 and CGI-2 amplified 5 CpG sites within the *Pappa* CGI, for a total of 10 sites in the island examined. For statistical analysis of methylation differences, the non-parametric Mann-Whitney U test was used, with a Bonferroni correction applied for multiple comparisons.

## Supporting Information

S1 Fig *Tnc* and *Tnfsf15* expression in mammary epithelial cells. *Tnc* (A) and *Tnfsf15* (B) expression was examined in MECs of *Mcs5c* susceptible and *Mcs5c* resistant rats at various ages. Gene expression relative to *Mcs5c* susceptible levels was determined via qPCR with *Tnc* and *Tnfsf15* standardized to *Tbp* expression. There were an average of n = 12 animals per group, and p-values were obtained using the non-parametric Mann-Whitney U test. Standard error bars are shown (*, P ≤ 0.05; **, P ≤ 0.01; ***, P ≤ 0.001).
(TIF)

S2 Fig Chromosome conformation capture profiles for additional *Pappa* bait regions. (A) A full 3C profile shows the relative interaction frequency (IF, y-axis) between the bait region P3-1 and regions spanning the entire *Mcs5c* locus in MECs at various ages. The x-axis indicates the distance between the tested region and P3-1 (UCSC Genome Browser, March 2012, rn5). (B) Three additional *Pappa* bait regions (P3-3, P4-1A, and P4-2; see Fig 4A for genomic locations) were tested for interactions with the *Mcs5c* TCE in MECs from 4 and 6 week old rats. In both (A) and (B), *Mcs5c* genotypes were combined within time points. (C) The *Pappa* bait region P3-3 was tested for interaction with the TCE in 4 and 7 week colon epithelial cells and 7 week liver hepatocytes. Only *Mcs5c* resistant rats were used in this analysis. For all graphs, multiple biological and technical replicates were used, and standard error bars are shown. P-values were obtained using the non-parametric Mann-Whitney U test (MEC–mammary epithelial cells; *, P ≤ 0.05; **, P ≤ 0.01; ***, P ≤ 0.001).
(TIF)

S3 Fig *Tnc* and *Tnfsf15* expression is unaffected by TCE knockdown. (A) *Tnc* and *Tnfsf15* expression in positive clones (n = 9) and WT LA7 cells (n = 3 independent cultures) was analyzed via qPCR and standardized to *Tbp* expression. P-values were obtained using the non-parametric Mann-Whitney U test, and standard error bars are shown. A scatterplot of *Tnc* (B) and *Tnfsf15* (C) expression versus *Mcs5c* copy number demonstrate no correlation between the two (Pearson correlation coefficient, R, = -0.229 & 0.229, n = 10 & 9, p-value = 0.524 & 0.553, respectively). A linear trend line is shown with the dotted line (slope = -0.121 & 0.173, respectively).
(TIF)

S1 Table Sequencing across target cut site of LA7 CRISPR clones. (XLSX)

S2 Table Sequencing of proximal target region of LA7 CRISPR clones. (XLSX)

S3 Table Sequencing of distal target region of LA7 CRISPR clones. (XLSX)

S4 Table MEC methylation levels of *Pappa* CpG island shore. (XLSX)

S5 Table MEC methylation levels of *Pappa* CpG island. (XLSX)

S6 Table Chromosome conformation capture (3C) primers. (XLSX)

S7 Table Sequencing primers and *Mcs5c* looping variants between the WF and WKy inbred rat strains. (XLSX)

S8 Table CRISPR gene editing primers. (XLSX)

S9 Table Custom made primers for bisulfite pyrosequencing methylation analysis. (XLSX)

S10 Table *In vitro* wild-type LA7 and CRISPR clone methylation levels. (XLSX)
