# Supplementary figures and images for: The Non-coding Mammary Carcinoma Susceptibility Locus, Mcs5c, Regulates Pappa Expression via Age-Specific Chromatin Folding and Allele-Dependent DNA Methylation
Source: PLoS Genet. 2016 Aug 18;12(8):e1006261. doi: 10.1371/journal.pgen.1006261 (PMC4990333; doi:10.1371/journal.pgen.1006261)

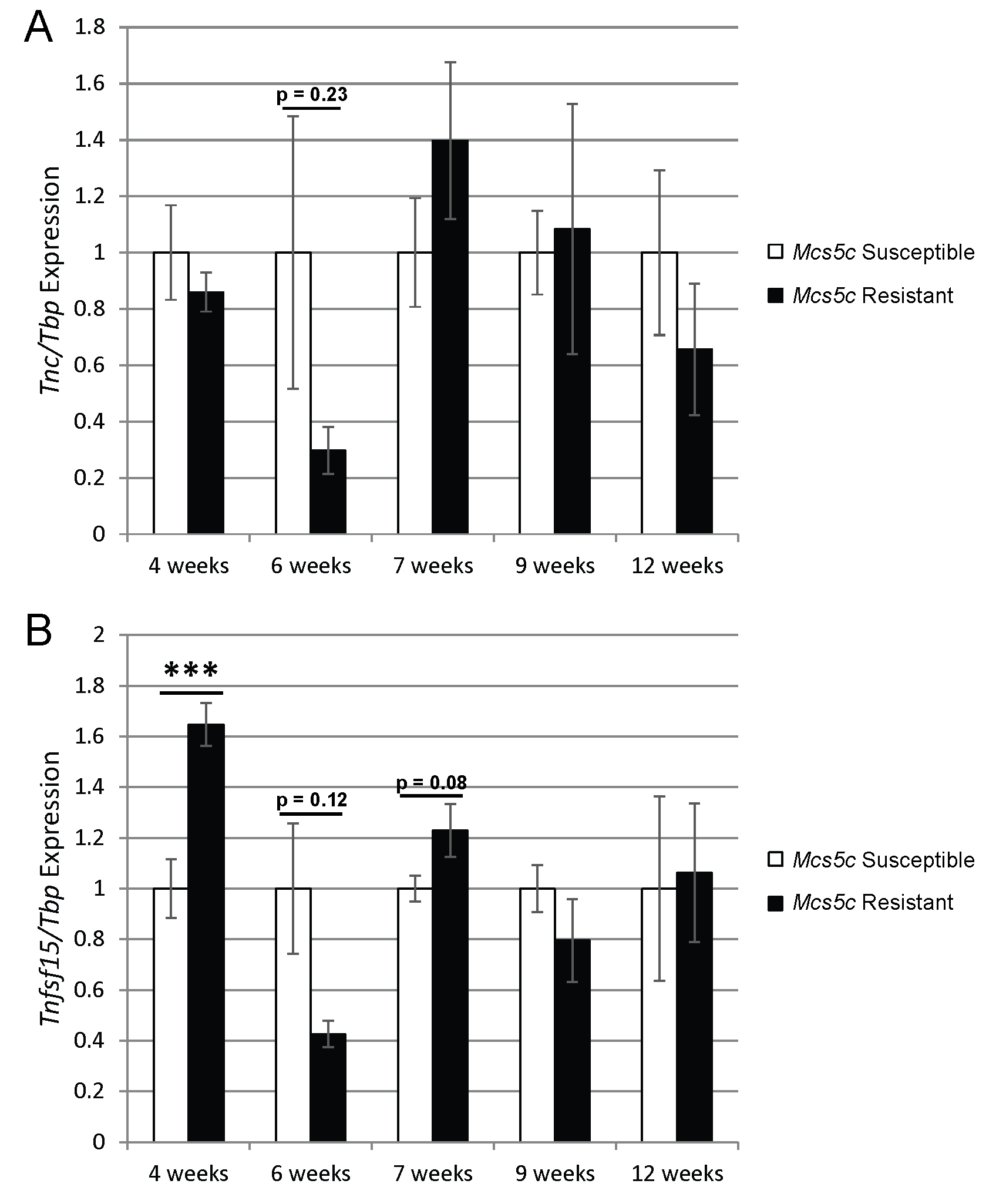

Supplement: S1 Fig — Tnc (A) and Tnfsf15 (B) expression was examined in MECs of Mcs5c susceptible and Mcs5c resistant rats at various ages. Gene expression relative to Mcs5c susceptible levels was determined via qPCR with Tnc and Tnfsf15 standardized to Tbp expression. There were an average of n = 12 animals per group, and p-values were obtained using the non-parametric Mann-Whitney U test. Standard error bars are shown (*, P ≤ 0.05; **, P ≤ 0.01; ***, P ≤ 0.001). (TIF) [file pgen.1006261.s001.tif]

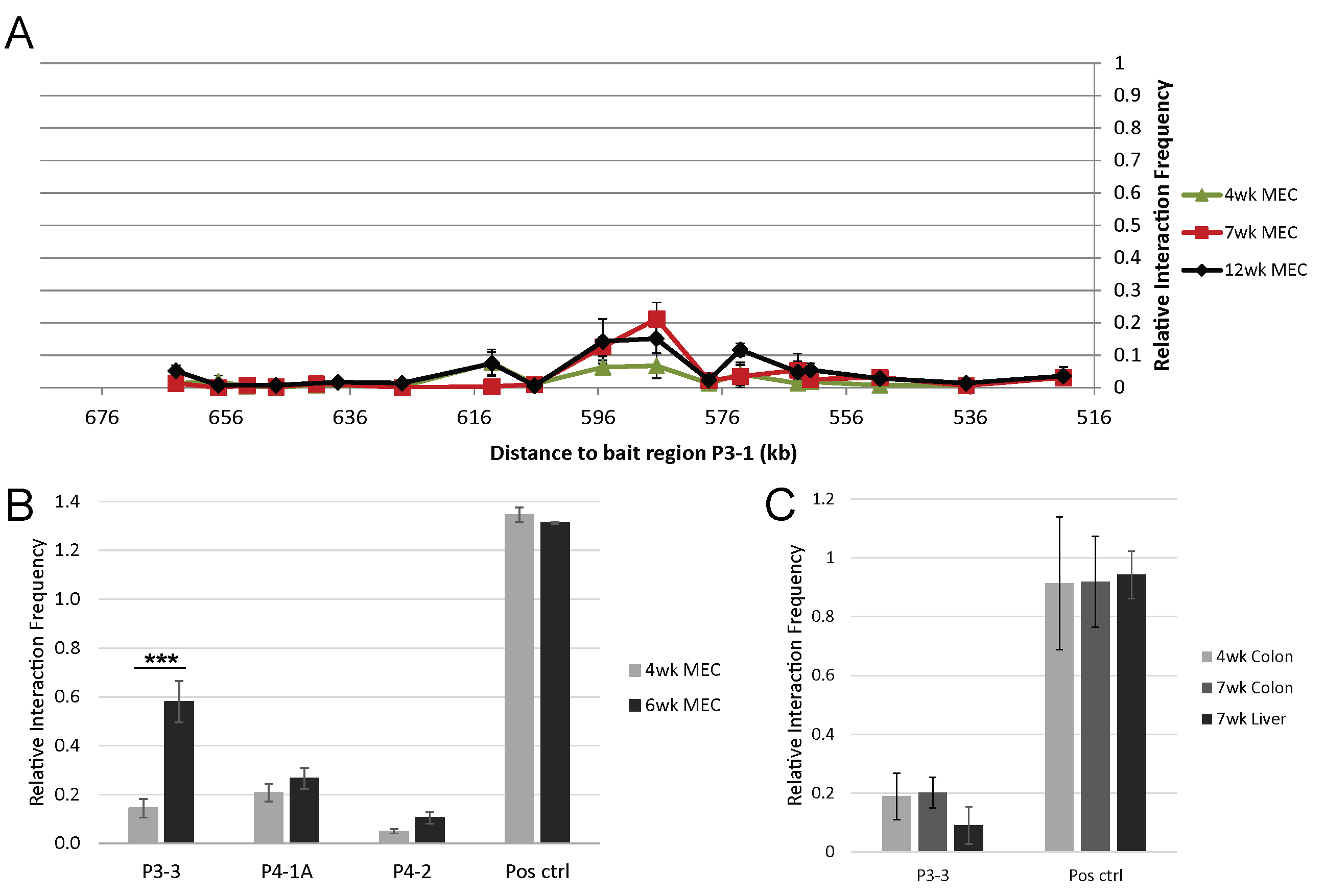

Supplement: S2 Fig — (A) A full 3C profile shows the relative interaction frequency (IF, y-axis) between the bait region P3-1 and regions spanning the entire Mcs5c locus in MECs at various ages. The x-axis indicates the distance between the tested region and P3-1 (UCSC Genome Browser, March 2012, rn5). (B) Three additional Pappa bait regions (P3-3, P4-1A, and P4-2; see Fig 4A for genomic locations) were tested for interactions with the Mcs5c TCE in MECs from 4 and 6 week old rats. In both (A) and (B), Mcs5c genotypes were combined within time points. (C) The Pappa bait region P3-3 was tested for interaction with the TCE in 4 and 7 week colon epithelial cells and 7 week liver hepatocytes. Only Mcs5c resistant rats were used in this analysis. For all graphs, multiple biological and technical replicates were used, and standard error bars are shown. P-values were obtained using the non-parametric Mann-Whitney U test (MEC–mammary epithelial cells; *, P ≤ 0.05; **, P ≤ 0.01; ***, P ≤ 0.001). (TIF) [file pgen.1006261.s002.tif]

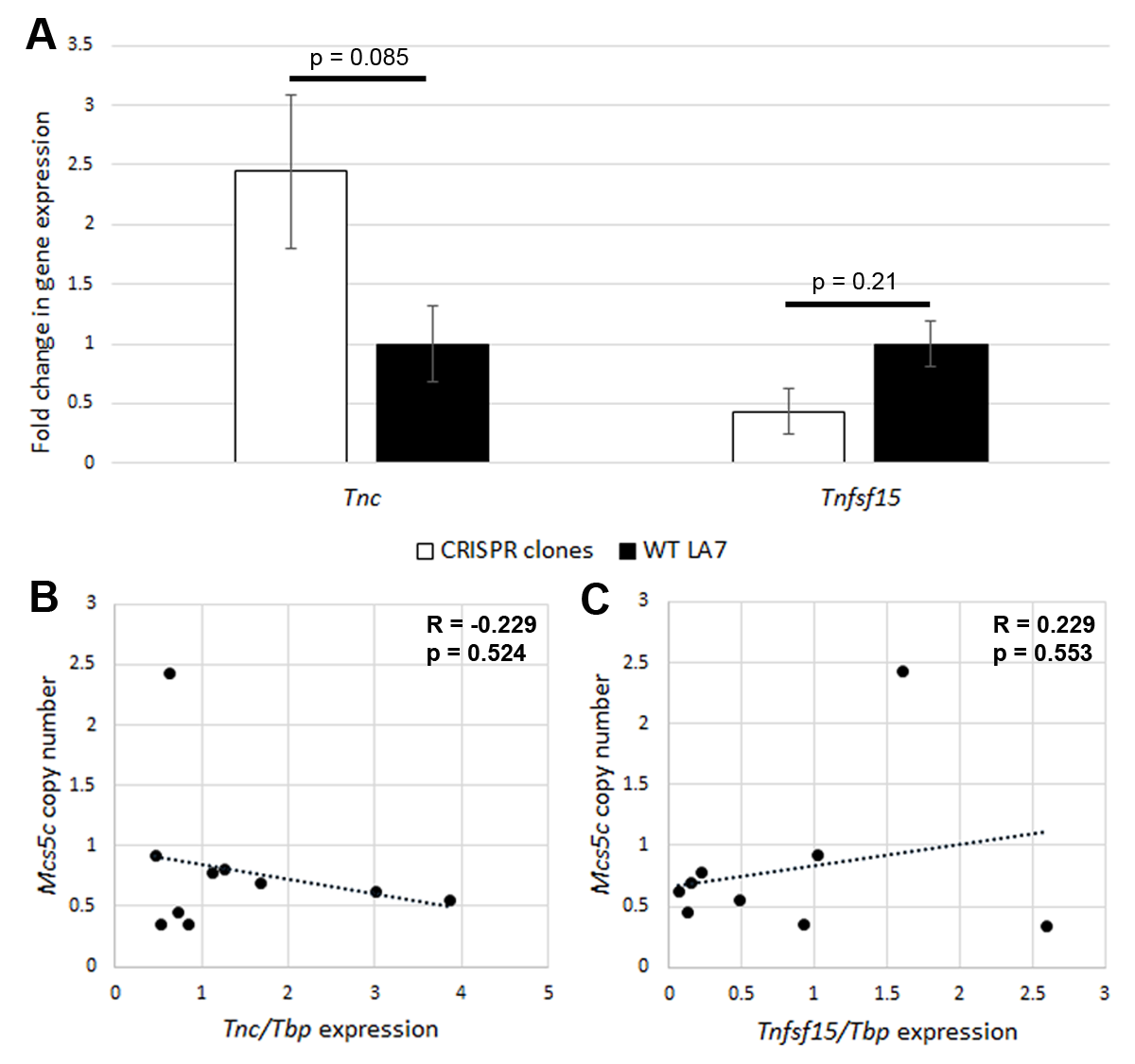

Supplement: S3 Fig — (A) Tnc and Tnfsf15 expression in positive clones (n = 9) and WT LA7 cells (n = 3 independent cultures) was analyzed via qPCR and standardized to Tbp expression. P-values were obtained using the non-parametric Mann-Whitney U test, and standard error bars are shown. A scatterplot of Tnc (B) and Tnfsf15 (C) expression versus Mcs5c copy number demonstrate no correlation between the two (Pearson correlation coefficient, R, = -0.229 & 0.229, n = 10 & 9, p-value = 0.524 & 0.553, respectively). A linear trend line is shown with the dotted line (slope = -0.121 & 0.173, respectively). (TIF) [file pgen.1006261.s003.tif]
